# P-775. Unlocking Synergistic Potency: Dual Action of β-Lactam and β-Lactamase Inhibitor in Combating *Mycobacterium abscessus* through β-Lactamase Inhibition and Multi-Target Receptor Inactivation

**DOI:** 10.1093/ofid/ofae631.969

**Published:** 2025-01-29

**Authors:** Eunjeong Shin, Khalid M Dousa, Christopher Bethel, Mary Nantongo, Steven M Holland, Eric Rubin, Jürgen B Bulitta, Barry N Kreiswirth, Robert A Bonomo

**Affiliations:** Case Western Reserve University, Lakewood, Ohio; VAMC, Cleveland, Ohio; Louis Stokes Cleveland VA Medical Center, Cleveland, Ohio; Case Western Reserve University, Lakewood, Ohio; National Institutes of Health, Bethesda, Maryland; Harvard School of Public Health, Boston, Massachusetts; University of Florida, Gainesville, Florida; Center for Discovery and Innovation, Hakensack Meridian Health, Nutley, New Jersey; Case Western Reserve University/ Louis Stokes Cleveland VA Medical Center, Cleveland, OH

## Abstract

**Background:**

*Mycobacterium abscessus* (*Mab*) poses significant clinical challenges, leading to chronic pulmonary disease in immunocompromised patients. Current treatment involve amikacin (AMK), which is toxic, highlighting the imperative for safer options. This study continues our evaluation of the synergistic effects of a β-lactam and β-lactamase inhibitor (BL/BLI) against *Mab* and to elucidate the underlying mechanism.

The inhibition constant (Ki,app)

**Figure:**
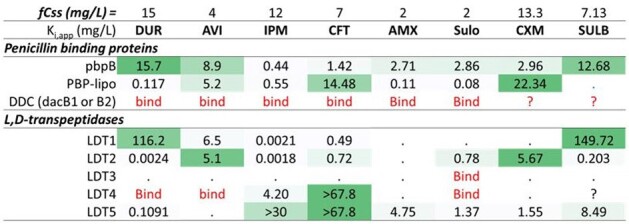

**Methods:**

The binding affinities and chemical interactions between target receptors (LDT1-5, DDC, PBP B, and PBP-lipo) and BL/BLI were determined via kinetics, mass spectrometry, DSF, flow cytometry and microscopy. The synergistic effects of BL/BLI were evaluated in time-kill studies, conducted over 10 days, using ATCC 19977 producing *Bla_Mab_*.

Time-kill curves of monotherapy or double β-lactams (A, D, and G) and their combinations with avibactam (B, E, and H) or durlobactam+sulbactam (C, F, and I).

**Figure:**
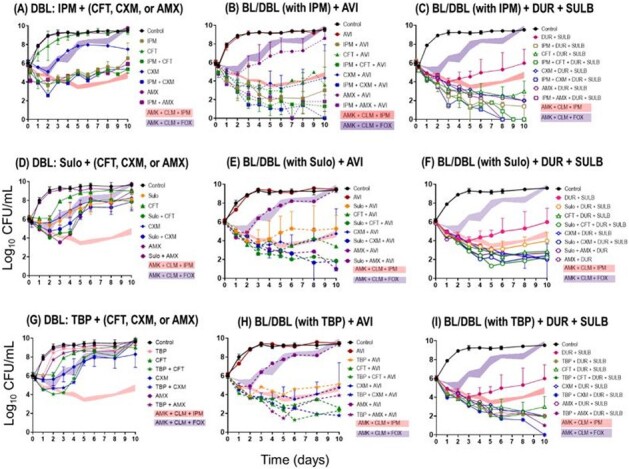

**Results:**

Imipenem (IPM) showed high binding affinities for both LDTs and PBPs, with an extremely low inhibition constant (*K_i,app_*; **Tab. 1**). Cephalosporins exhibited moderate binding affinity for both LDTs and PBPs, whereas amoxicillin (AMX) selectively targeted PBPs. The inactivation of *Bla_Mab_* and LDTs/PBPs by durlobactam (DUR) exhibited greater efficacy compared to avibactam (AVI), aligning with their respective bactericidal effects. DUR showed a 75-fold lower *K_i,app_* for *Bla_Mab_* in comparison to AVI. The *K_i,app_* of DUR for PBP B, PBP-lipo, and LDT2 fell below the clinical concentration, whereas those of AVI did not. Single β-lactam treatment resulted in minimal killing (∼1 log_10_ reduction), but adding AVI significantly enhanced killing by inhibition of β-lactamase (∼4 log_10_ reduction for IPM+AVI and ∼2 log_10_ reduction for others; **Fig. 1**). DUR alone showed 2 log_10_ reduction, and when combined with IPM or dual BL, it achieved near-eradication, surpassing the SOC (AMK + Clarithromycin + IPM or cefoxitin). Inhibition of PBP-lipo by sulopenem, IPM, DUR, and AMX changed the cell morphology to filaments. In accordance with the binding affinity to LDTs and PBPs, alterations in melting temperature were detected.

**Conclusion:**

IPM + DUR + SUL showed the most killing of *Mab* with limited regrowth. This outcome can be attributed to β-lactamase inhibition and the inactivation of multiple targets, not only LDTs but also PBPs. This combination strategy holds promise for enhanced efficacy of *Mab* treatment, necessitating further study in clinical trials.

**Disclosures:**

**All Authors**: No reported disclosures

